# The relationship between time to surgical debridement and incidence of infection in grade III open fractures

**DOI:** 10.1007/s11751-012-0130-y

**Published:** 2012-03-31

**Authors:** Jagwant Singh, Rohit Rambani, Zaid Hashim, Raghu Raman, Hemant Kumar Sharma

**Affiliations:** Trauma and Orthopaedics, Hospital Hull Royal Infirmary, Anlaby Road, Hull, HU3 2JZ UK

**Keywords:** Grade III open fractures, Debridement, Infection rate

## Abstract

**Objective:**

The purpose of this study was to determine the association between time to initial debridement and infection rate in high-energy (grade III) open fractures of tibia.

**Methods:**

All patients presenting with open fractures were included in the study. The inclusion criteria were Gustilo III A, B and C open fractures of tibia. Time of injury, time of arrival to the hospital, time of initial debridement and subsequent soft tissue procedures were recorded. The primary outcome measure was a diagnosis of infection or osteomyelitis at 1 year. Secondary outcome measure was fracture union at 1 year.

**Results:**

Sixty-seven (67) patients with grade III open fractures were included; the mean age was 32.4 years (54 males and 13 females). Eight patients (12 %) in this study went on to develop a deep infection, and there were 6 (8.4 %) non-unions. The infection rate for patients in the group who underwent debridement in less than 6 h and those greater than 6 h was 13.1 and 10.8 %, respectively. No statistically significant difference could be demonstrated between the two groups (*p* = 0.56). While there was no significant relationship between grade of fracture and infection rate (*p* = 0.07), the relationship between grade of fracture and non-union was significant (*p* = 0.02).

**Conclusion:**

Our study shows that the risk of developing an infection was not increased if the primary surgical management was delayed more than 6 h after injury. Therefore, reasonable delays in surgical treatment for patients with open fractures may be justified in order to provide an optimal operating environment.

## Introduction

Open tibial fractures pose a major treatment challenge for surgeons. Infection and non-union are the main complications after open diaphyseal fractures of the tibial [1–7]. The reported rate of infection is between 5 and 50 % and that of non-union between 7 and 60 % [1–6, 8–10]. In fractures complicated by infection or non-union, there will invariably be secondary procedures [3, 5, 6, 8–10]. A thorough debridement, washout and skeletal stabilization are the mainstays of initial treatment along with intravenous antibiotics. Recent studies have advocated the role of early wound coverage as a means to decrease wound infection [3, 4, 11]. The urgency for debridement has not been clearly defined with mostly surgeons advocating within the first 6 h [12–14].

The origin of the 6-h rule is unclear. Friedrich, in his experiments on guinea pigs in 1898, reported lower infection rates when debridement was performed within 6 h [15]. Robson later confirmed this on human wounds [16]. The practice of treating open fractures within 6 h can result in complex operations being performed out of normal working hours by relatively inexperienced surgeons, anaesthetists and theatre staff with potentially sub-optimal results [12, 13].

We conducted this study to evaluate the association between time to initial surgical debridement and rates of infection in open fractures of the tibia.

## Materials and methods

All patients presenting to our hospital with Grade III open fractures of the tibia between 1997 and 2008 were included in the study. Approval from the local audit, research and development department was obtained. Data were collected retrospectively. The inclusion criteria were patients with Gustilo III A, B and C open fractures of tibia. Patients were excluded if they had a spinal cord injury, the presence of infection prior to the injury or a coincident full thickness burn. Patients who presented later than 24 h after the injury, referrals from other hospitals and those who were followed up in centres other than the study centre were also excluded.

The patients had been reviewed by the orthopaedic team upon presentation at the accident and emergency department (A&E). Management included assessment of wound, removal of large contaminants, a photograph of open wound, cover with occlusive dressings and an assessment of the distal neurovascular status. All patients with clean wounds received intravenous antibiotics of either 1.5 g cefuroxime (Cefuroxime Axetil) or 1.2 g of Co-amoxyclav. Intravenous analgesia (Morphine) and tetanus prophylaxis was given as per immunization status. The patients were prepared for surgery (debridement and stabilization) immediately after initial presentation. The limiting factors to immediate surgery were associated comorbidities in the patient, grade of surgeon attending and emergency theatre access.

At surgery, all patients underwent wound debridement and fracture stabilization. Fractures were classified using the Gustilo and Anderson classification system [10, 17]. Plastic surgeons were involved in the early assessment, and the timing of subsequent debridement, definitive fixation and soft tissue procedures were decided jointly.

Patients received intravenous antibiotics for 48 h or longer depending on wound contamination, extent of debridement, surgeon assessment and timing of wound closure. Demographic data including the time of injury, time of presentation to the hospital, time of initial debridement and subsequent soft tissue procedures were noted. Comorbidities including diabetes and smoking were also noted. The patients were divided into two groups; those who had debridement within 6 h of injury (<6 h) and those who waited for more than 6 h (>6 h).

The primary outcome measure was a diagnosis of infection or osteomyelitis up to 1 year. The patients were followed up regularly up to clinical and radiological fracture union or until a definitive procedure for infection or non-union had been carried out with a minimum subsequent follow-up of at least 1 year. Superficial infection that was adequately managed with a course of antibiotics was not included in our analysis. Pin tract infection (associated with external fixator use) not requiring surgery was also excluded.

The hypothesis was time to initial debridement does not influence the rate of infection in grade III open tibial fractures. Any evidence of wound infection led to culture swabs being taken and patients treated with a prolonged duration of intravenous antibiotics and, if needed, surgical intervention. Advice from a microbiologist was sought early. Patients who had wound infections confirmed by culture results were treated with prolonged antibiotics.

## Results

Sixty-nine patients with grade III open fractures satisfied the inclusion criterion, out of which 67 were included in the study. One patient was transferred to another hospital for further treatment and was therefore excluded. Another died from an unrelated illness prior to fracture union.

**Fig. 1:**
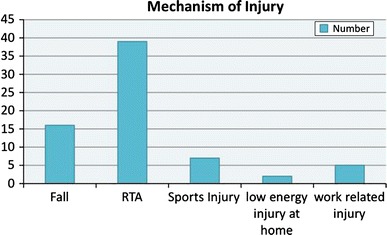
Mechanism of injury

**Fig. 2:**
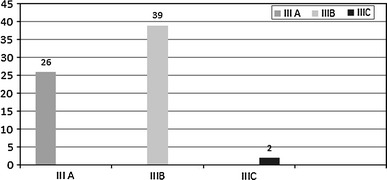
Grades of fracture (Gustillo and Anderson classification)

**Fig. 3:**
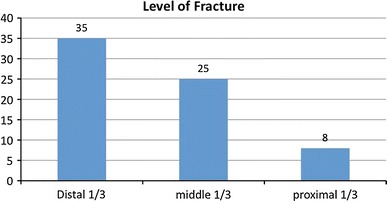
Level of fracture

**Fig. 4:**
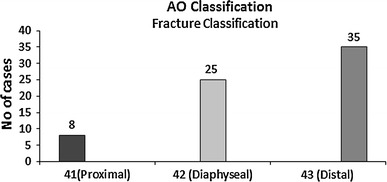
AO classification

The mean age was 32.4 years (7–89); we had 54 male and 13 female patients. Road traffic accident (RTA) was the most common mechanism of injury (56.5 %). Figure 1 shows the distribution of mechanism of injury. There were 26 grade III A fractures (39 %), 39 grade III B fractures (58 %) and 2 grade III C (3 %) as shown in Fig. 2. The majority of the patients in our study had fractures of the distal third of the tibia (Figs. 3, 4).

The time from injury to surgery ranged from 2 h 35 min to 12 h with an average time of 5 h 40 min. Sixty-nine per cent of patients had their wound debridement within 6 h of presentation to the hospital. However, this reduced to 55 % when time from injury to debridement was taken into consideration. The major reason for the delay was lack of prompt access to theatre.

The infection rate for patients who underwent debridement in less than 6 h was 13.1 %. Two patients, who had debridement within 6 hours, developed pin tract infection (away from original fracture site), were treated by oral antibiotics and needed no further intervention. If these two cases of pin tract infection were included, the infection rate in this <6 h group is 18.4 % (Table 1). In contrast, the infection rate in those who underwent debridement after 6 h group was 10.8 %. The results were analysed using Fisher’s exact test and no statistically significant difference could be demonstrated between the two groups (*p* = 0.560).

**Table 1:** Infection and non-union in the two groups

Time delay to debridement	No	Infection	Non-union	United without infection
<6 h	38	7 (18.4 %)^a^	5	33
>6 h	29	3 (10.8 %)	1	26

^a^This includes two cases of pin tract infection, which were managed successfully with oral antibiotics, and no further surgical intervention was required

Further regression analysis of timing of surgery revealed no statistically significant increase in infection rates for 3, 6 or 9 h.

Eight patients (12 %) developed a deep infection. There were 6 (8.4 %) non-unions. Of the eight with deep infection, five were from the <6 h group and three from the >6 h group.

Grade III B fractures had the highest rate of infection and non-union at twenty and thirteen per cent, respectively. When the grade of fracture was paired with infection, the *p* value was not statistically significant (*p* = 0.07, paired sample *t* test). When grade of fracture was paired to non-union, the*p* value was statistically significant (*p* = 0.02, Table 2).

**Table 2:** Association between various grades of fracture and infection and non-union rate (*p* value <0.05 is significant)

Fracture	III A (*N* = 26)	III B (*N* = 39)	III C (*N* = 2)	*p* value
Infection	0	8	0	0.07
Non-union	1	5	0	0.02
Delayed union	2	1	0	
Pin tract infection/superficial infection	0	2	0	

There was no increase in infection rate in those treated after 6 h compared with those treated within 6 h.

## Discussion

The key elements in managing open fractures of tibia include prompt and appropriate antibiotics, debridement, skeletal stabilization and early soft tissue cover [18]. Debridement is an important surgical step in the management of open tibial fractures. Much evidence is provided in the literature to support early debridement. The experimental studies by Friedrich [15] and Robson [19] showed the infection threshold of 10^5^ organisms per gram of tissue was reached in at an average of 5.17 h [16]. Clinical studies by Kreder and Armstrong, Kindsfather and Jonassen have supported the importance of the 6-h rule [14, 16, 20]. However, it should be noted that studies by Friedrich and Robson were carried out in pre-antibiotic era [19].

There are studies [12, 13, 21–25] that challenge the 6-h rule for open tibial fractures; these have found no difference between early and delayed treatment groups with respect to the overall infection rate. However, studies by Skaggs, Sungaram, Harley and Al-Arabi have emphasized the importance of prophylactic antibiotics [6, 20–22]. Early administration of antibiotics is now considered a standard of care [1–3, 7, 18]. A recent Cochrane systematic review showed that the administration of antibiotics after an open fracture reduces the risk of infection by 59 % [26]. Our results show that there is no increased risk of infection if first debridement is delayed beyond the 6-h threshold. All the patients in our study received prophylactic antibiotics in emergency department as a part of primary management.

The average rate of infection in grade III fractures is 10–50 % depending on the severity of the injury [1, 7, 10, 27, 28]. The overall infection rate in this study was 14.9 % (10 cases), decreasing to 12 % if the two cases of pin tract infection were excluded. All these patients had Gustilo III B injuries. There were two cases of grade III C fracture, which were operated within 6 h of presentation; both underwent urgent debridement, external fixation and posterior tibial artery reconstruction (Table 3).

**Table 3:** Various fixation techniques

Treatment	III A	III B	III C
External fixator	11	20	2
Intra-medullary Nailing	9	12	
Plating	4	4	
Others (Steinman pin, POP)	2	3	
Total	26	39	2

Debridement in grade III open fractures needs to be performed by an experienced surgeon. Naique et al. [29] reported an association between adequacy of debridement and infection and suggested that adequacy of the debridement, rather than timing, determines outcome.

Our study suggests that there is no association between time to debridement and infection rate. It may be prudent to delay definitive debridement so that better treatment by experienced surgeons and staff familiar with equipment can be provided. In a busy District General or peripheral hospital, where immediate access to theatre for all such injuries may not be possible, delaying debridement for some injuries may not influence the outcome. The limitation of our study is that it is a retrospective case–control study. A power analysis by Skaggs et al. [23] revealed that 11,390 cases would be needed in order to detect a 20 % difference in infection rate.

Skeletal stabilization should be performed at the time of initial debridement [3, 30, 31]. This can be achieved by intramedullary nails, plates or external fixators depending on the morphology of the fracture. Restoring alignment of the limb eliminates gross movement at the site of fracture, limits further soft tissue damage and decreases the risk of further bacterial spread [30, 31]. Nearly half of the patients (46 %) in our study were treated with an external fixator and 30 % by an intramedullary nail.

We suggest the 6-h rule must be interpreted with caution. In a level 1 trauma centre, it may be possible to gain prompt theatre access to allow this rule to be satisfied. However, this and other studies have indicated that the infection rate is not associated with a reasonable delay to debridement, especially if the delay is to facilitate treatment by the appropriate senior surgical personnel. Our study also recognizes the importance of a multi-disciplinary management protocol as recommended by the recent BOA/BAPRAS (British Orthopaedic Association/British Association of Plastic, Reconstructive and Aesthetic surgeons) standards for management of open fractures [18].

## Conclusion

This study suggests that infection rate is not increased if the primary surgical debridement was delayed more than 6 h after injury. Reasonable delays in surgical treatment for patients with open fractures may be justified in order to provide an optimal operating environment.
